# An L-theanine derivative targets against SARS-CoV-2 and its Delta and Omicron variants

**DOI:** 10.1016/j.heliyon.2022.e09660

**Published:** 2022-06-09

**Authors:** Jing Lu, Ying Zhang, Dan Qi, Chunyan Yan, Benhao Wu, Jason H. Huang, Jianwen Yao, Erxi Wu, Guoying Zhang

**Affiliations:** a Key Laboratory of Molecular Pharmacology and Drug Evaluation, Ministry of Education, Collaborative Innovation Center of Advanced Drug Delivery System and Biotech Drugs in Universities of Shandong, School of Pharmacy, Yantai University, Yantai, Shandong, 264005, China; b Shandong YingdongYinghao Biotechnology Inc., Yantai, Shandong, 264670, China; c Department of Pharmaceutical Sciences, North Dakota State University, Fargo, ND, 58105, USA; d Neuroscience Institute, Baylor Scott & White Health, Temple, Texas, 76502, USA; e Department of Pharmacy, Yantai Yuhuangding Hospital (Laishan branch), Yantai, Shandong, 264003, China; f College of Medicine, Texas A&M University, College Station, TX, 77843, USA; g College of Irma Lerma Rangel College of Pharmacy, Texas A&M University, College Station, TX, 77843, USA; h LIVESTRONG Cancer Institutes and Department of Oncology, Dell Medical School, The University of Texas at Austin, Austin, TX, 78712, USA

**Keywords:** TBrC, SARS-CoV-2, M^pro^/3CL, ACE2, Mutant spike proteins, NF-κB

## Abstract

Recent research efforts have shown that tea has activities against SARS-CoV-2. However, the active compounds and the action mechanisms are largely unknown. Here we study the inhibitory potential of L-theanine from tea and its semi-synthesized derivative, a small-molecule fluorescent compound, ethyl 6-bromocoumarin-3-carboxylyl L-theanine (TBrC) against infection and replication of SARS-CoV-2 and the underlying mechanisms of action. We reveal that TBrC has potential activities against SARS-CoV-2 in addition to its activity against lung cancer. TBrC showed extracellular inhibition of SARS-CoV-2 M^pro^/3CL and the host cell receptor ACE2 while interacting with the viral spike glycoproteins (wild-type, Delta, and Omicron mutants). Moreover, TBrC and L-theanine significantly suppressed growth and TNFα-induced nuclear transcriptional activation of NF-κB in human lung cancer cells without affecting the viability of normal lung cells, suggesting a potential protection of TBrC and L-theanine from pulmonary damages in SARS-CoV-2 infected patients, especially for lung cancer patients with SARS-CoV-2 infection.

## 1 Introduction

SARS-CoV-2 (severe acute respiratory syndrome coronavirus 2), identified as the causative agent of a new respiratory syndrome, has rapidly spread throughout the world, causing an ongoing pandemic with millions of deaths (Karim and Karim, 2021; Worldometer, 2021). Although several vaccines obtained emergency use authorization (EUA) or approval from health authorities worldwide and are being used, many people are reluctant to get vaccinated and the emergence of viral escape mutants including the mutant Omicron SARS-CoV-2 may make vaccines less effective (Centers for Disease Control and Prevention CDC, 2020; Ferre et al., 2021; Kannan et al., 2022; Zhang et al., 2021). Thus, there is an urgent need of continued efforts for new treatment options for SARS-CoV-2.

L-theanine (T) is a unique amino acid existing in tea plants, the mushroom *Xerocomus badius*, and some species of genus *Camellia*. It has been marketed in the United States as a diet supplement and granted GRAS (generally recognized as safe) status by FDA. Clinical research efforts have shown that L-theanine attenuates the adverse effects of anticancer drugs and improves the quality of life in patients with colorectal cancer (Tsuchiya et al., 2016). We have also previously reported that L-theanine inhibited migration and invasion of human lung cancer cells (Liu et al., 2009). Recently, due to the SARS-CoV-2 pandemic, researchers reported that tea has antiviral activities against SARS-CoV-2 (Chowdhury and Barooah, 2020; Liu et al., 2021; Ohgitani et al., 2021). However, the active compounds and the action mechanisms have not been clarified and studied in detail. We semi-synthesized an L-theanine derivative ethyl 6-bromocoumarin-3-carboxylyl L-theanine (TBrC) and confirmed that TBrC displayed much stronger inhibition than L-theanine on cell migration and induction of cell apoptosis in highly-metastatic lung cancer and cervical cancer cells (Ji et al., 2014; Liu et al., 2016).

Considering the lung cancer patients infected with SARS-CoV-2 showing an increasing mortality (Rolfo et al., 2022) and the challenge of emerging variants such as Delta and Omicron as well as tea's antiviral activity against SARS-CoV-2, in this study, we probe the possibility of L-theanine and its derivative TBrC against mutant Delta and Omicron SARS-CoV-2. Our results suggest the potential of TBrC as a new treatment for the management of SARS-CoV-2 variants as well as the molecular mechanisms of action.

## 2 Materials & methods

### 2.1 Chemicals and antibodies

The TNFα was purchased from R&D systems, Inc. (Shanghai, China). Trans AM NF-κB p65 Transcription Factor Assay kit (Catalog No. 40096) was purchased from Active Motif North America (Carlsbad, CA 92008). Bay 11–7082 (Bay), DMSO, and all other chemicals including materials for the synthesis of TBrC were acquired from Sigma-Aldrich (Shanghai, China). The cell lines of human NSCLC A549 and NCI–H460 (H460) as well as normal human embryonic lung fibroblast (MRC-5) were obtained from the American Type Culture Collection. The 3-(4,5-dimethylthiazol-2-yl)2,5-diphenyl-tetrazolium bromide (MTT), SARS-CoV-2 M^pro^/3CL and ACE2 Inhibitor Screening Kits (M^pro^/3CL Kit Catalog No. P0315M; ACE2 Kit Catalog No. P0320M) were obtained from Beyotime Biotechnology, Inc. (Shanghai, China).

### 2.2 *In vivo* fluorescent signals of TBrC in mice

Animal experiments were performed with the ethical approval by the Institutional Animal Care & Use Committee (IACUC) of Yantai University (China). Female BALB/c *nu/nu* mice and C57BL/6 mice (age range, 6 weeks) were purchased from Beijing Vital River Laboratory Animal Technology Co., Ltd. (Beijing, China). All animal procedures were carried out in accordance with the guidelines established by the IACUC at Yantai University, China. TBrC (30 mg/kg, intraperitoneal [i.p.]) or L-theanine (30 mg/kg, i.p.) as a control was injected into the mice. Three hours later, the fluorescent imaging *in vivo* was recorded under 530 nm excitation and 600 nm emission. Images were captured on a Kodak Image Station 4000 Multi-Modal Imaging System (IS4000MM) equipped with an X-ray unit and on a Kodak Image Station 2000 (Carestream Health, Rochester, NY, USA.).

### 2.3 Molecular docking

The Surflex-Dock program in Sybyl-X 2.1.1 software (Tripos, Inc., St. Louis, MO, USA) was used to identify possible binding modes of TBrC and four viral targets (M^pro^/3CL, ACE2-wildtype spike, ACE2-Deltaspike, and ACE2-Omicronspike). The 3D structure of TBrC was generated and optimized using Tripos force field and Gasteiger-Huckel charges by the minimize module. The 3D structure of M^pro^/3CL (PDB ID: 7JU7) (Drayman et al., 2021) was used for docking, and the active site was defined as the binding pocket of masitinib in the crystal structure. The site for RBD of wildtype spike bound to ACE2 (PDB ID: 6M0J) sets around hotspots Asn487, Lys417, Gln493, Tyr505, Tyr449, Thr500, Asn501, Gly446, Tyr489, Gly502 in RBD in spikes and Gln24, Asp30, Glu35, Glu37, Asp38, Tyr41, Gln42, Tyr83, Gln325, Glu329, Asn330, Lys353, Arg393 in ACE2 (Lan et al., 2020).The Delta (bearing mutations L452R and T478K) and Omicron RBDs (K417N, S477N, T478K, E484A, N501Y) were mutated at the desired position with Biopolymer module of Sybyl. Default settings for ligand-protein docking were used throughout the simulations. The 2D plots are generated using LIGPLOT v2.2.4 (Laskowski and Swindells, 2011).

### 2.4 Molecular dynamics (MD) simulation

To investigate the stable states of ligand-protein bindings, four independent simulations were carried out for TBrC interacted with these four viral targets. The initial coordinates of TBrC in the proteins were derived from the above-mentioned docking conformations, and simulations were performed with GROMACS 5.1.4 (Abraham et al., 2015). Taking the TBrC-M^pro^/3CL simulation as an example, first the protein was modeled with AMBER99SB-ILDN (Lindorff-Larsen et al., 2010). A GAFF force field was applied for TBrC using the program antechamber in AMBER14 (Case et al., 2014), and the parameters were converted to GROMACS format using the amb2gmx.pl script (Mobley et al., 2006). The system was solvated with TIP3P waters and the charges were neutralized with 0.15 M NaCl. Then, energy minimization for the system was carried out using the steep-descent algorithm for 50,000 steps. The canonical ensemble by heating the system from 0 K to 300 K was performed using velocity rescaling (Bussi et al., 2007) and the isothermal–isobaric ensemble (P = 1 bar and T = 300 K) was conducted by the Parrinello-Rahman barostat(Nosé and Klein, 1983; Parrinello and Rahman, 1981) for 100 ps, respectively. Finally, a 70 ns production run for ligand-protein was performed. The root-mean-square deviation (RMSD) and root-mean-square fluctuation (RMSF) values calculated from the MD trajectory were used to verify the stability and changes of the ligand-protein complex, which have been widely used for the binding of the compounds against SARS-CoV-2 targets (Bhowmik et al., 2021a, 2021b; Mishra et al., 2021). Moreover, a cluster protocol based on the RMSD of the conformations using the GROMOS clustering algorithm (Daura et al., 1999) was used to extract the representative conformation from the dynamically equilibrated MD trajectory.

### 2.5 Fluorescence resonance energy transfer (FRET) assay

The enzyme activity of SARS-CoV-2 ACE2 and M^pro^/3CL was determined by FRET assay using SARS-CoV-2 M^pro^/3CL (Catalog No. P0315M) and ACE2 (Catalog No. P0320M) Inhibitor Screening Kits (Beyotime Biotechnology, Inc. Shanghai, China) according to the manufacturer's instructions. The kit materials mainly include SARS-CoV-2 M^pro^/3CL enzyme having the same amino acid sequence as the 2019 CoV-2 M^pro^/3CL or ACE2 enzyme, M^pro^/3CL or ACE2 substrate, ebselen or MLN-4760 as the positive control of M^pro^/3CL or ACE2 inhibitor, respectively, M^pro^/3CL or ACE2 assay buffer. The negative control DMSO (vehicle) showed 100% of enzyme activity of ACE2 and M^pro^/3CL without inhibition. The detailed material and method information on these two kits can be found at the links below: https://www.beyotime.com/product/P0312M.htm for M^pro^/3CL, and https://www.beyotime.com/product/P0320M.htm for ACE2.

For the activity assay of TBrC, 93 *μ*L reaction volume containing 1 *μ*M SARS-CoV-2 ACE2 or M^pro^/3CL in the presence or absence of TBrC, L-theanine (T), or the positive control MLN-4760 (for ACE2) or ebselen (for M^pro^/3CL) was pre-incubated at room temperature for 20 min. The reaction was initiated by addition of the 2 *μ*M fluorescent protein substrate and monitored continuously for 5-20 min at 393 nm (ACE2) or 490 nm (M^pro^/3CL) after excitation at 325 nm (ACE2) or 340 nm (M^pro^/3CL), respectively, using a Synergy H1 microplate reader (BioTek Instruments, Inc., Winooski, VT, USA). The concentrations of TBrC used in the FRET assays of SARS-CoV-2 ACE2 and M^pro^/3CL were from 0.37 *μ*g/mL to 90 *μ*g/mL with three-fold series dilution. The inhibitory rate of TBrC and L-theanine against SARS-CoV-2 ACE2 and M^pro^/3CL were determined by using six different concentrations of TBrC and L-theanine.

### 2.6 Cell culture and the *in vitro* cell growth assay

For the *in vitro* experiments, the cell lines of human lung cancer A549 and H460 as well as normal MRC-5 cells were cultured in DMEM (Sigma, Co., MO) supplemented with 10% heat-inactivated fetal bovine serum (FBS), glutamine (2 mM), penicillin (100 U/mL), and streptomycin (100 *μ*g/mL) at 37 °C in a humidified incubator with 95% air/5% CO_2_ atmosphere. The relative cell growth rate (%) was measured at 48 h after the treatments using MTT assay according to our previously reported methods (Ji et al., 2014; Liu et al., 2016). Briefly, cells were planted in 96-well plates (Becton Dickinson, NJ, U.S.A.) at 2 × 10^3^/well and incubated overnight to allow attachment. The cells in control group (0) were treated with DMSO [0.1% (v/v), final concentration]. The cells were incubated in DMEM medium supplemented with 10% FBS containing different concentrations of TBrC (5–100 *μ*g/mL), or Bay11-7082 (Bay, 0.62 *μ*g/mL, a specific NF-*κ*B inhibitor as the positive control). After 48 h of treatment, the absorbance values in each test group were measured using MTT assay. The relative cell growth (%) was calculated based on the absorbance of sample vs. the absorbance of the control (vehicle). Each experiment was repeated three times.

### 2.7 NF-κB p65 transcription factor assay

A549 cells were planted in 6-well plates (Becton Dickinson, NJ, U.S.A.) at 8 × 10^5^ cells/well and incubated overnight to allow attachment. The cells in control group (0) were treated with DMSO [0.1% (v/v), final concentration]. A549 cells were treated for 30 min with TBrC (50 *μ*g/mL) and L-theanine (50 *μ*g/mL) as well as Bay (0.62 *μ*g/mL) as a positive control. After 6 h of TNFα (20 ng/mL) treatment, the treated cells were then pelleted by centrifugation at 1,000 rpm for 5 min at 4 °C and re-suspended in ice-cold buffer A (10 mM HEPES (pH 7.9), 10 mM KCl, 0.1 mM EDTA, 1 mM DTT, 0.5 mM phenylmethysulfonyl fluoride (PMSF), 1 *μ*g/mL leupeptin, 5 *μ*g/mL aprotinin). Following the addition of 25 *μ*L 10% NP40, the suspension was vortexed and centrifuged at 14,500 rpm for 1 min at 4 °C; the supernatant was designated as the cytoplasmic fraction. Nuclei were re-suspended in 50 μL of ice-cold buffer B (20 mM HEPES (pH 7.9), 0.4 M NaCl, 1 mM EDTA, 1 mM DTT, 1 mM PMSF, 25% glycerol, 1 *μ*g/mL leupeptin, 5 *μ*g/mL aprotinin) and centrifuged at 14,500 rpm for 5 min. The supernatant was used as the nuclear fraction and protein concentration determined by the Bradford method. The nuclear protein was used to detect the NF-κB p65 activity (%). The TNFα induced the nuclear transcriptional activation of NF-κB p65 (NF-κB p65 activity/%) in human lung cancer A549 cells was determined by a NF-κB p65 transcriptional factor assay kit (Trans AM NF-κB p65 Catalog No. 40096), according to the manufacturer's instructions. The detailed material and method information on the kit can be found at the links below. https://www.activemotif.com/catalog/216/transam-nf%CE%BAb-p50-p52-p65-family-kits.

### 2.8 Statistical analysis

Data were expressed as mean ± standard deviation (S.D.) and analyzed by the SPSS 16.0 software to evaluate the statistical difference. Statistical analysis was done using the Analysis of Variance (ANOVA) and Bonferroni post-test. Values between different treatment groups were compared. The relative cell growth (%) and NF-κB activity (%) are shown for each group. For all tests, *p* values less than 0.05 were considered statistically significant. All statistical tests were two-sided.

## 3 Results

### 3.1 TBrC exhibits potential activities against SARS-CoV-2 M^pro^/3CL

**Figure 1:**
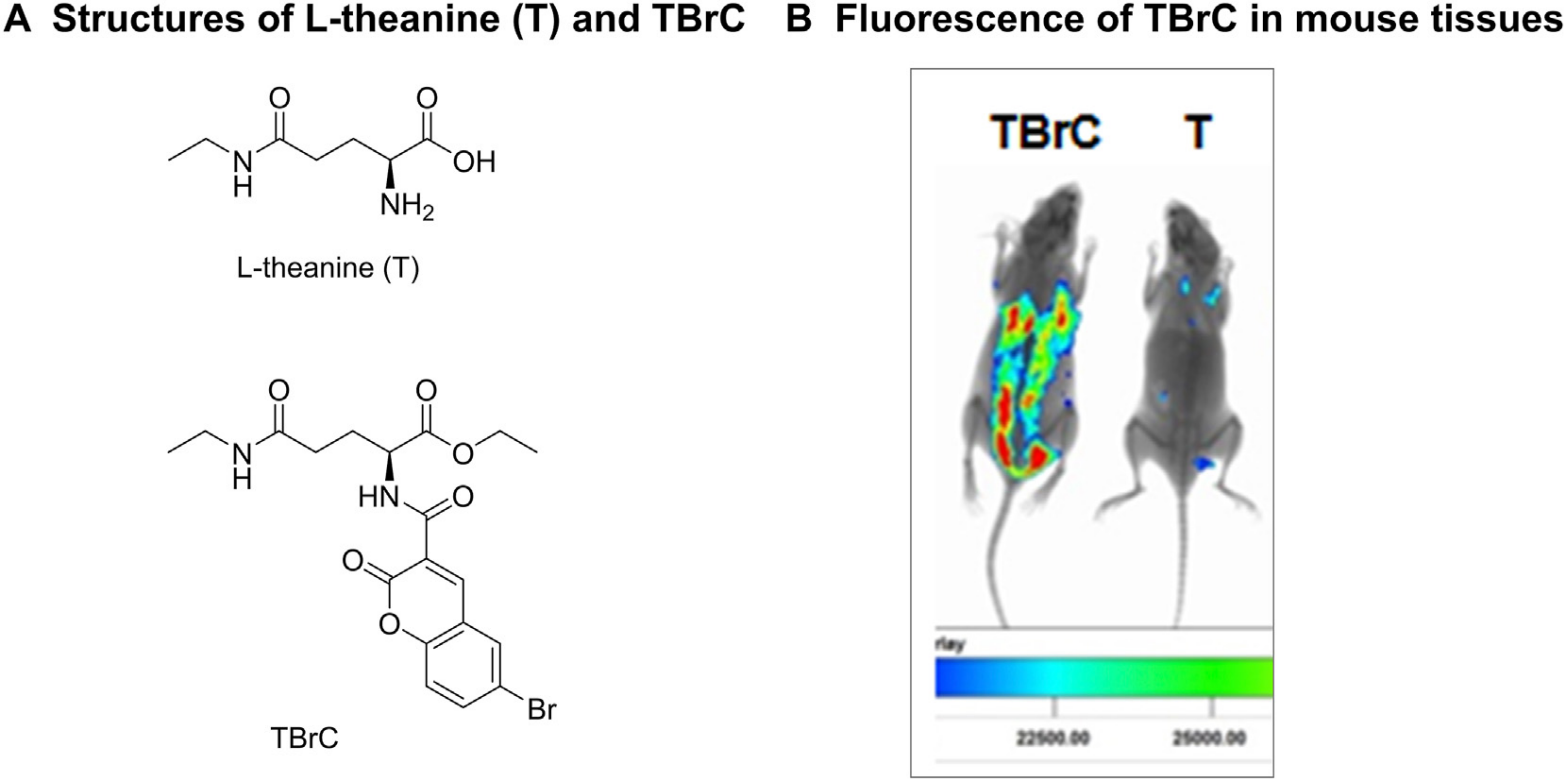
Chemical structures of L-theanine (T) and TBrC as well as the fluorescence of TBrC displayed in mouse tissues (3 h after intraperitoneal injection of TBrC at 30 mg/kg).

In our previous studies, L-theanine (T) derivative TBrC exhibited a much stronger suppression than T against highly-metastatic lung cancer and cervical cancer cells (Ji et al., 2014; Liu et al., 2016). The chemical structures of T and TBrC are shown in Figure 1A. In this study, we show that TBrC displayed a strong fluorescent signal in mouse lungs and other tissues 3 h after intraperitoneal injection, and the fluorescence of TBrC clearly demonstrate its *in vivo* distribution (Figure 1B).

**Figure 2:**
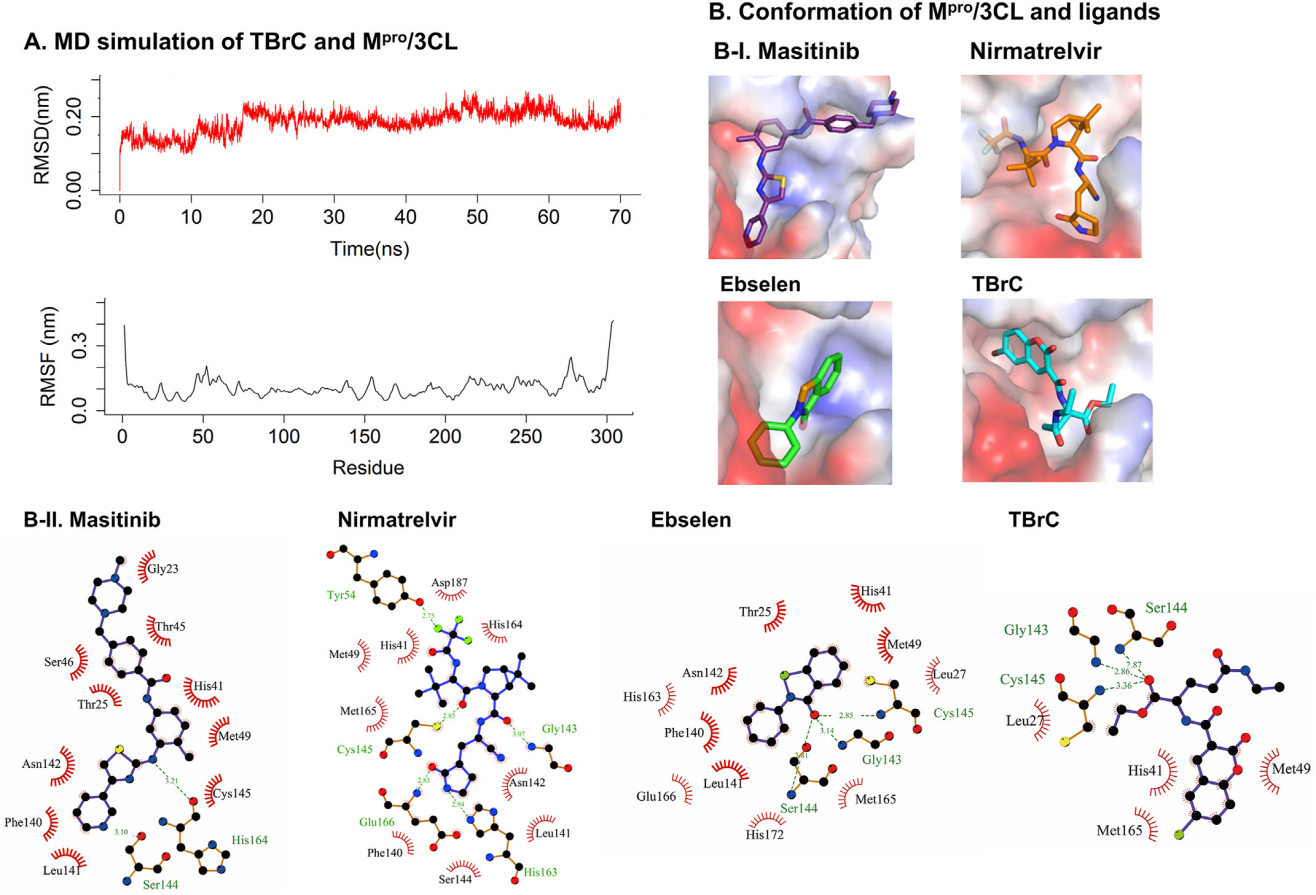
TBrC exhibits potential activities against SARS-CoV-2 M^pro^/3CL. (A) Molecular dynamics (MD) simulation of TBrC and M^pro^/3CL. RMSD (red) and RMSF (black) values of M^pro^/3CL are calculated from the backbone after least squares fit to backbone. (B) Binding modes of TBrC and positive control inhibitors interacting with M^pro^/3CL. The protein surface is colored by its electrostatic potential, from red (-51.710) to blue (51.710). The ligands are shown in purple (masitinib), orange (nirmatrelvir), green (ebselen), and cyan (TBrC). In 2D plots, hydrogen bonds and hydrophobic interactions are shown as green dashed lines and red “eyelashes”, respectively.

In order to further develop the activity against SARS-CoV-2 and understand the underlying mechanism of action of T and TBrC, we studied the inhibitory potential of T and TBrC against SARS-CoV-2 and explored the molecular mechanisms. Molecular docking analysis indicated that the positive control inhibitor masitinib (an anticancer drug) locates in the catalytic region of M^pro^/3CL by forming noncovalent interactions with key catalytic residues His41 and Cys145 in catalytic site (Figure 2B) (Menendez et al., 2020), which is almost identical with the ligand conformation in the crystal structure (PDB ID: 7JU7) (Drayman et al., 2021). Other two inhibitors ebselen and nirmatrelvir showed similar binding modes with M^pro^/3CL (Figure 2B). The binding mode of TBrC and M^pro^/3CL was analyzed by molecular docking and further molecular dynamics (MD) simulations (Figure 2A, B). The RMSD value of M^pro^/3CL showed a stable equilibrium after 17 ns, and the RMSF plot indicated an average atomic fluctuation <0.15 nm for amino acid residues, verifying the conformational stability of protein-ligand complex. The representative conformation (frame-53600) characterizing 51.5 % conformations in MD clustering analysis with a cutoff of 0.12 nm (Figure 2A) indicated that TBrC also firmly located in the binding site of the target by a network of hydrogen and hydrophobic interactions (Figure 2B). TBrC's branched chains form three H-bonds with M^pro^/3CL, including one between the ester's oxygen to the nitrogen atom of the key catalytic residue Cys145. The coumarin moiety of TBrC occupies the S2 binding pocket of the protease, forming a hydrophobic interaction with His41, the second residue in the protease catalytic dyad. Similar binding mode of TBrC with three positive controls indicate that TBrC acts likely as a competitive inhibitor of M^pro^/3CL.

### 3.2 TBrC potentially inhibits the interactions of ACE2 and wildtype, Delta or Omicron mutant spike proteins of SARS-CoV-2

**Figure 3:**
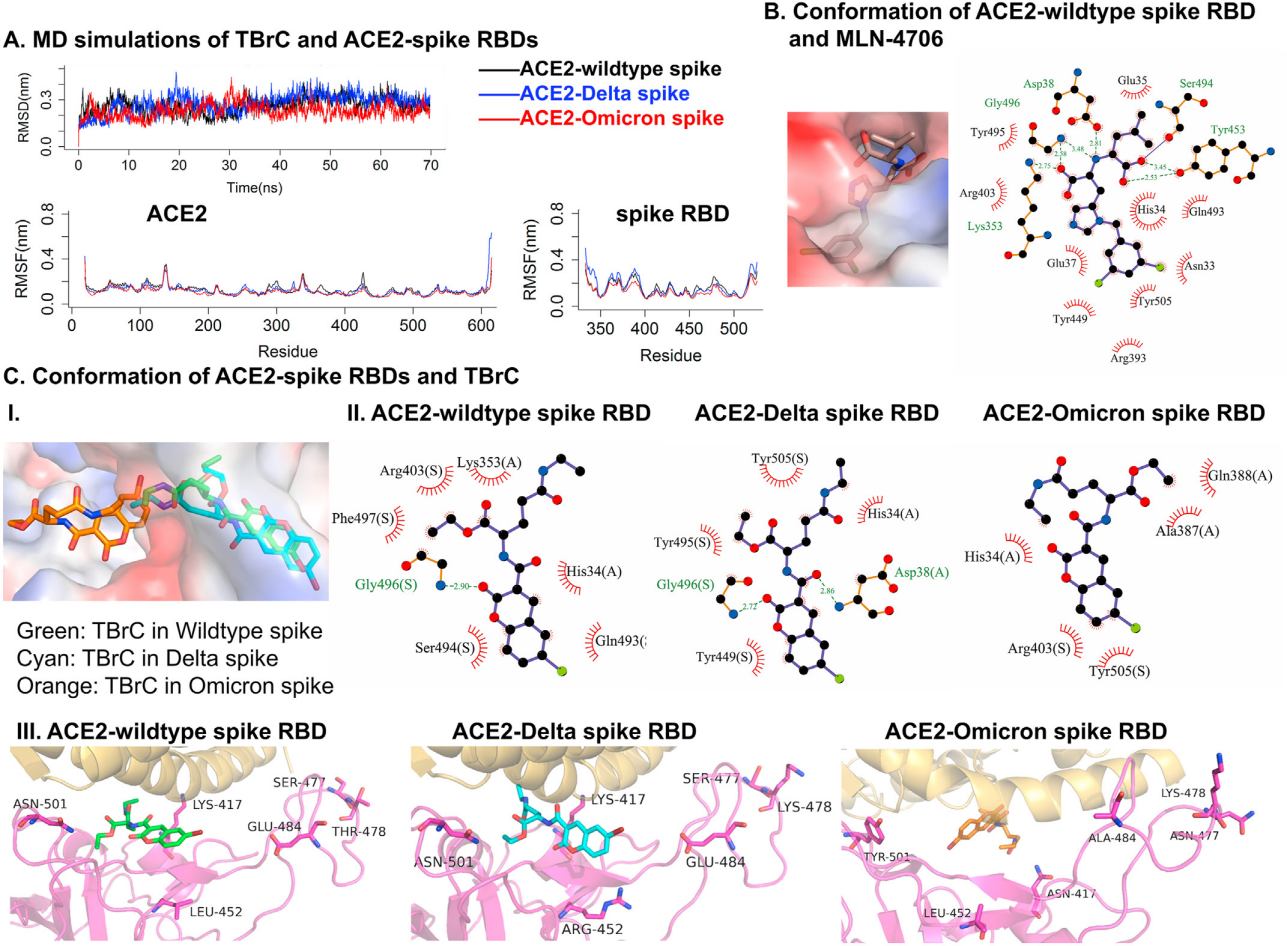
TBrC potentially inhibits the interactions of ACE2 and wildtype, Delta or Omicron mutant spike proteins of SARS-CoV-2. (A) MD simulations of TBrC bound to ACE2 and wildtype spike RBD (black), Delta spike RBD (blue), and Omicron spike RBD (red). (B) Binding modes of MLN-4760 with ACE2-wildtype spike RBD. (C) Binding modes of TBrC with ACE2-spike RBDs. The labels are shown in magenta cartoon and sticks (key residues and spike RBDs), golden cartoon (ACE2), green, cyan and orange sticks (three TBrCs in wildtype, Delta and Omicron targets), respectively. The protein surfaces are colored by their electrostatic potentials, from red (-66.271) to blue (66.271) in wildtype ACE2-spike RBDs, from red (-65.180) to blue (65.180) in Delta variant, from red (-64.019) to blue (64.019) in Omicron variant, respectively. In 2D plots, (A) and (S) mean the interactions of TBrC bound to ACE2 and RBDs of spikes, respectively.

Next, we investigated the possibility of TBrC interfering with the receptor-binding domain (RBD) in wildtype, Delta, and Omicron mutant spikes bound to ACE2 (PDB ID: 6M0J) (Lan et al., 2020) by MD simulations. The stable trajectories of the TBrC bound to ACE2-spike RBDs after 10 ns and the low fluctuations of the residues signify that the ligand is stabilized with the molecular interactions at the binding pocket of the ACE2-spike RBDs (Figure 3A). The representative conformations (frame-51300 for ACE-wildtype spike and frame-65950 for ACE2-Delta spike) indicated that TBrC showed similar bindings due to no direct interactions with the residues in position 452 and 478 of spike associated with Delta mutants (Figure 3C), whose conformation is similar with MLN-4760 bound to ACE2-spike RBDs (Figure 3B) (Nami et al., 2022). The branched theanine groups of TBrC are stabilized by hydrogen bond with Gly496 in spike RBDs as well as hydrophobic interactions with His34 in ACE2, respectively (Figure 3C). The coumarin moiety of TBrC forms hydrophobic interactions with multiple residues in spike RBDs and ACE2. In contrast, Omicron spikes with more mutants have weak interactions for TBrC, making hydrophobic contacts with Tyr505 in spike RBD and His34 in ACE2. We consider that this may be because the Omicron mutant has important amino acid mutation sites of four other mutant strains (Alpha, Beta, Gamma, and Delta spikes), including sites that enhance cellular receptor affinity and viral replication capacity. These results indicate that TBrC potentially has stronger inhibitory effects on wild type and Delta mutant spikes than Omicron spike bound to ACE2. It is possible to enhance the TBrC's activity by further optimizing, such as introducing some branched groups in the coumarin ring or long chain in theanine branch, for destroying the interaction of ACE2-spike RBD.

### 3.3 Extracellular inhibition of SARS-CoV-2 M^pro^/3CL and ACE2 activities by TBrC

**Figure 4:**
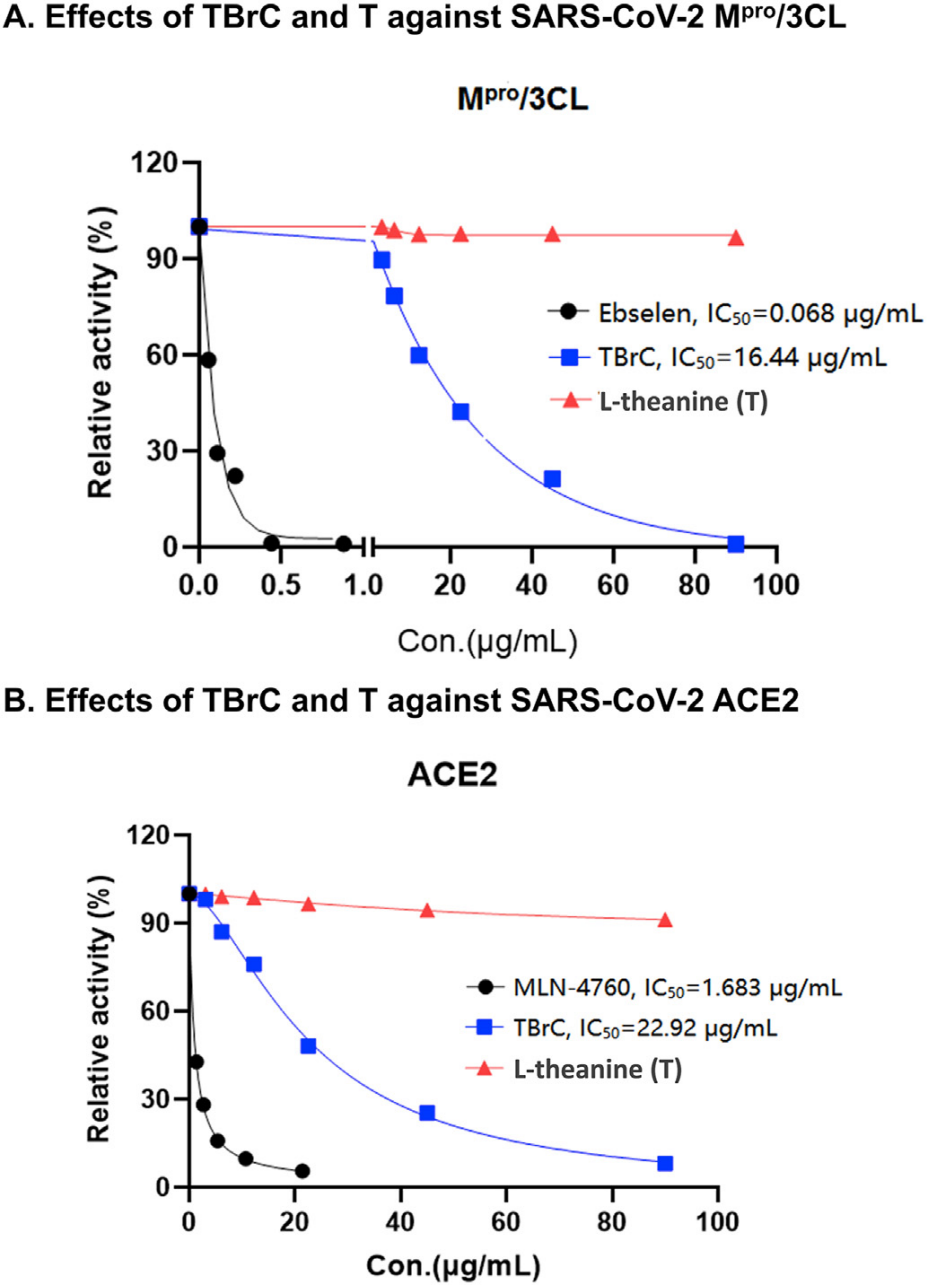
Extracellular inhibition of SARS-CoV-2 M^pro^/3CL and ACE2 activities by TBrC. TBrC exhibited the extracellular inhibition of activities of SARS-CoV-2 M^pro^/3CL (A) and ACE2 (B) at the indicated concentrations, but T displayed no significant inhibition. The well-known small molecule inhibitors of M^pro^/3CL (Ebselen) and ACE2 (MLN-4760) as the positive controls show remarkable suppression of M^pro^/3CL (A) and ACE2 (B) activities.

In order to further confirm the inhibitory activity of TBrC against SARS-CoV-2 M^pro^/3CL and ACE2, we confirmed by Fluorescence Resonance Energy Transfer (FRET) that TBrC (0.37 *μ*g/mL to 90 *μ*g/mL) showed the extracellular inhibition of SARS-CoV-2 M^pro^/3CL activity in a dose-dependent manner (IC_50_ = 16.44 *μ*g/mL, Figure 4A), indicating that M^pro^/3CL is a potential target for TBrC. The positive control ebselen, a well-known specific inhibitor of M^pro^/3CL (Jin et al., 2020), showed also strong inhibition of M^pro^/3CL (IC_50_ = 0.068 *μ*g/mL, Figure 4A), but T has no inhibitory activity for M^pro^/3CL. Furthermore, the results of FRET assay showed that TBrC (0.37 *μ*g/mL to 90 *μ*g/mL) dose-dependently suppressed the extracellular activity of ACE2 (IC_50_ = 22.92 *μ*g/mL, Figure 4B), although T did not display significant repression of ACE2. The positive control MLN-4760, a well-known specific inhibitor of ACE2 (Dales et al., 2002), also exhibited significant inhibition of ACE2 activity (IC_50_ = 1.683 *μ*g/mL, Figure 4B).

### 3.4 TBrC and L-theanine (T) significantly suppress the growth of human NSCLC A549 and H460 cell lines as well as the A549 cell NF-κB activity without affecting cell viability of human normal lung MRC-5 cells

**Figure 5:**
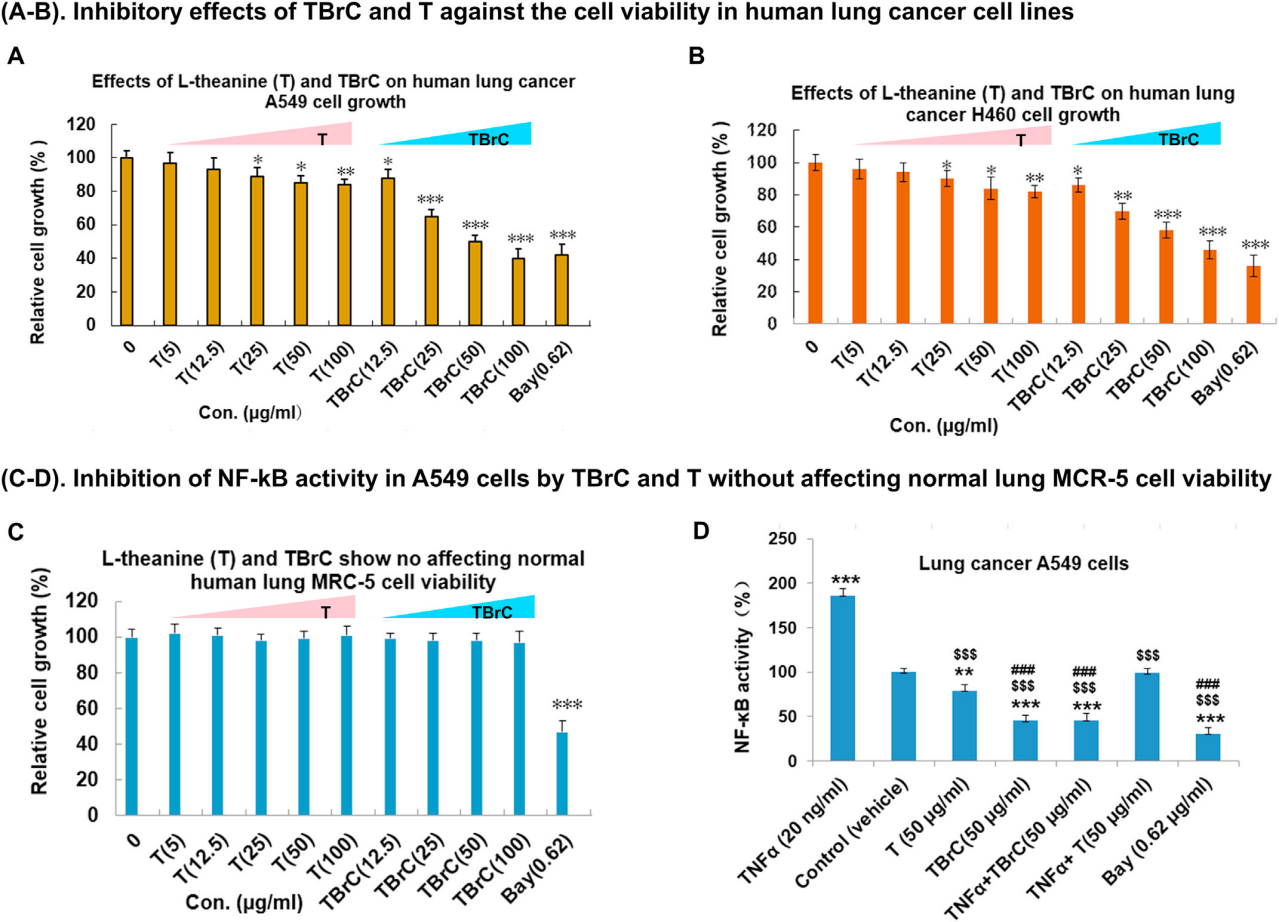
TBrC and L-theanine (T) suppressed the growth of human lung cancer A549 and H460 cells as well as host nuclear transcriptional activation of NF-κB p65 in A549 cells without affecting the cell viability of normal human embryonic lung fibroblasts (MRC-5 cells). (A–C) TBrC and T displayed significant growth inhibition of lung cancer A549 (A) and H460 (B) cell lines without affecting the cell viability of normal human lung MRC-5 cells (C) at the indicated concentrations (48 h treatment), but Bay11-7082 (Bay/0.62 *μ*g/mL, a specific inhibitor of NF-κB) significantly suppressed the normal MRC-5 cell growth. (D) The TNFα (20 ng/mL) induced the nuclear transcriptional activation of NF-κB p65 in human lung cancer A549 epithelial cells was effectively inhibited by TBrC (50 *μ*g/mL), T (50 *μ*g/mL) and Bay (0.62 *μ*g/mL), determining by the Trans AM NF-κBp65 Transcription Factor Assay Kit. ∗*p* < 0.05, ∗∗*p* < 0.01, ∗∗∗*p* < 0.001 compared to control group; ^$$$^*p* < 0.001, compared to TNFα group; ^###^*p* < 0.001, compared to L-theanine (T) group or TNFα + L-theanine (T) group.

Next, we performed MTT assay and demonstrate that TBrC and T at concentrations of 5-100 *μ*g/mL dose-dependently suppressed the growth of human non-small cell lung cancer (NSCLC) A549 and H460 cell lines (Figure 5A, B) without affecting cell viability of the normal human embryonic lung fibroblast MRC-5 cells (Figure 5C) after 48 h treatment of these cell lines with the same concentrations of TBrC and T, although Bay11-7082 (Bay/0.62 *μ*g/mL, a specific inhibitor of NF-κB) exhibited toxicity to the normal MRC-5 cells by suppressing the cell growth (Figure 5A, C). We previously reported that TBrC induced the cell apoptosis and inhibited tumor growth in highly-metastatic Lewis lung cancer (Ji et al., 2014). In addition, by using NF-κB p65 transcription factor assay, we further found that TBrC, T (50 *μ*g/mL) and a positive control Bay (0.62 *μ*g/mL) also inhibited TNFα-induced nuclear transcriptional activation of NF-κB p65 in human lung cancer A549 cells (Figure 5D). TBrC displays much stronger inhibition of the growth and NF-κB p65 activity in human lung cancer cells, compared to T (Figure 5).

**Figure 6:**
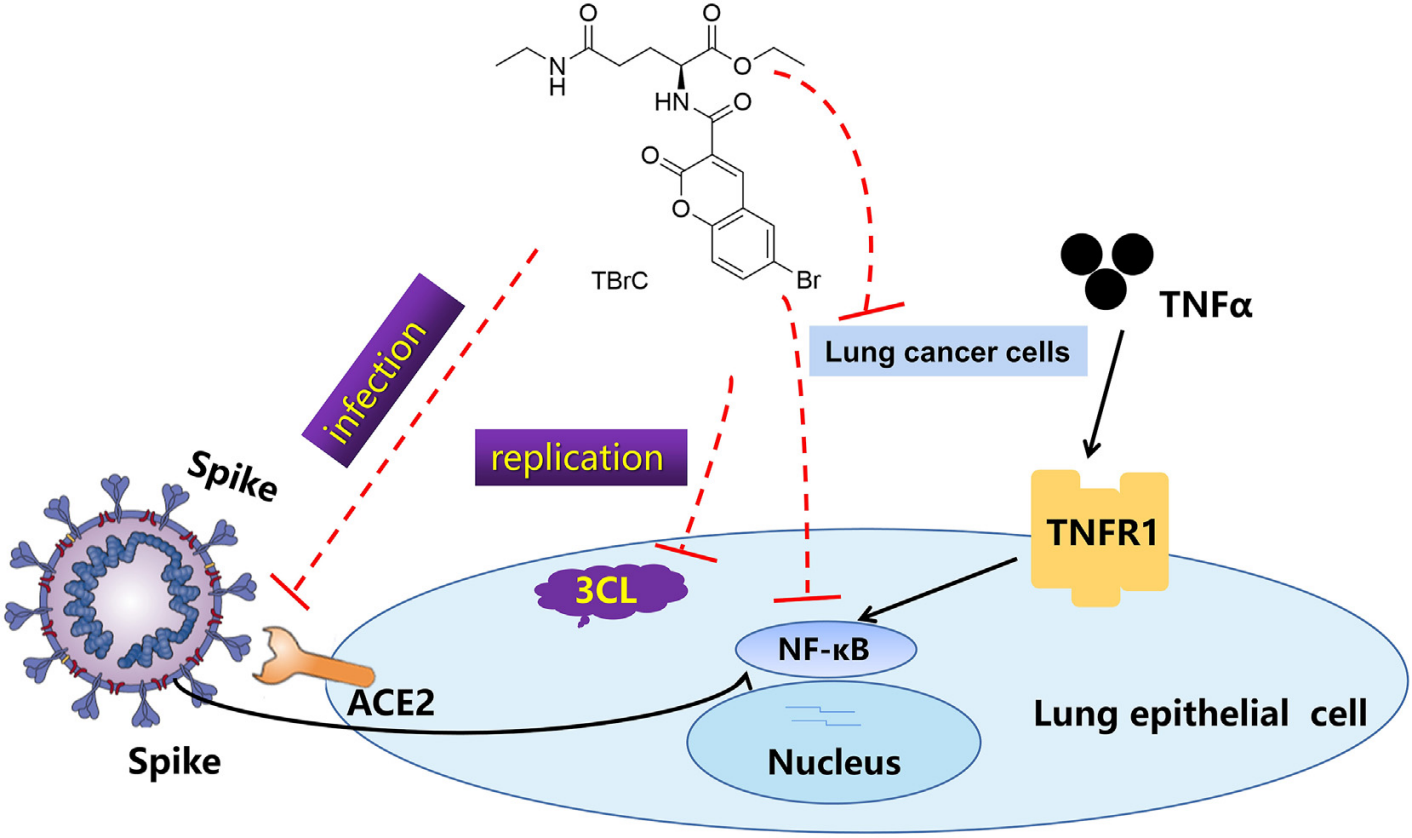
The possible underlying molecular mechanisms of action of TBrC against SARS-CoV-2.

Based on our findings mentioned-above, the effects of TBrC on SARS-CoV-2 M^pro^/3CL, host cell receptor ACE2 and spike proteins and human lung cancer cells, the molecular mechanisms underlying TBrC's action are summarized in Figure 6.

## 4 Discussion

The ongoing pandemic has 500 million cases and 6.19 million deaths, making it one of the deadliest in history (WHO, 2022). SARS-CoV-2 vaccines play a role in curbing spreading of the viruses. However, some people are resistant or hesitant to be vaccinated and more variants such as Omicron SARS-CoV-2 may cause current vaccines ineffective. Omicron is markedly resistant to neutralization by serum not only from patients who recovered from SARS-CoV-2, but also from individuals who were vaccinated with one of the four widely used SARS-CoV-2 vaccines (Liu et al., 2022). Patients with lung cancer are especially vulnerable to SARS-CoV-2 with a greater than seven-fold higher rate of becoming infected with SARS-CoV-2, a greater than three-fold higher hospitalization rate with high complication rates, and an estimated case fatality rate of more than 30%. The reasons for the increased vulnerability are not known (Rolfo et al., 2022). It is reported that binding and live-virus neutralizing antibody titers to SARS-CoV-2 mRNA vaccines in NSCLC patients were lower than the healthy vaccinees, with significantly lower live-virus neutralization of Delta, and more importantly, the Omicron variant compared to the wild-type strain. This result highlights the concern for cancer patients given the rapid spread of SARS-CoV-2 Omicron variant (Valanparambil et al., 2022). Therefore, cancer patients appearing vulnerable to SARS-CoV-2 infection and having poor outcomes may be partly due to host genetic factors and dysregulation of SARS-CoV-2-required genes (Huang et al., 2021). There is a report based on the data analysis results showing that more transcript variants (i.e., alternative splicing [AS] events are found in SARS-CoV-2 infected lung cancer A549 cells than in mock-treated cells; the transcript variants are enriched in important biological pathways that were not detected in the studies, suggesting the pathways may lead to new molecular mechanisms of SARS-CoV-2 pathogenesis (Sun et al., 2021). It may exist the complications associated with COVID variants and progression of lung cancer. Our present study on the molecular mechanism of TBrC against SARS-CoV-2 could help further to understand the interacting relationship between SARS-CoV-2 infection and progression of lung cancer and to accelerate the process of developing new simultaneous effective agents against SARS-CoV-2 and lung cancer.

In the present study, we show that TBrC has the potential of suppressing the main protease M^pro^/3CL of SARS-CoV-2 and ACE2 activities as well as interacting with M^pro^/3CL, wildtype spike, Delta mutant spike, and Omicron spike bound to ACE2 (Figures 2, 3, and 4). M^pro^/3CL, which is critical for viral replication of SARS-CoV-2, is a key target for therapeutic development (Drayman et al., 2021; Jin et al., 2020). SARS-CoV-2 virus entry into host cells is mediated by interaction between the spike protein and the host cell ACE2 receptor (Barnes et al., 2020; Yang et al., 2020). The wildtype spike and Delta mutant spike as well as mutant Omicron spike play important functions during SARS-CoV-2 infection since SARS-CoV-2 infects host cells using its spike glycoprotein to bind ACE2. During SARS-CoV-2 infection, spike increases DNA binding of NF-κB p65. NF-κB p65 is one of the key transcription factors stimulating peripheral blood mononuclear cells (PBMCs) and lung epithelial cells to release proinflammatory cytokines such as IFN, TNFα, etc., which causes pulmonary damages (Olajide et al., 2021; Su et al., 2021; Yin et al., 2021). For lung cancer, TNFα enhances the nuclear transcriptional activation of NF-κB p65 in human lung cancer A549 cells (Figure 5D), which could further promote proliferation, migration, invasion, progression and/or metastasis, involving in change of gene expressions and genetic variations in lung cancer and other cancer as reported previously from our and other laboratories (Athie et al., 2020; Ji et al., 2014; Liu et al., 2016; Tang et al., 2006; Yang et al., 2015). Thus, enhanced NF-κB p65 activity could increase, by a way of positive feedback, both lung cancer progression and SARS-CoV-2 infection with quickening the virus replication and/or genetic variation.

Our findings from this study may partly explain the challenging issues of current widespread concern mentioned-above summarized as follows: why SARS-CoV-2 infected lung cancer A549 cells have more transcript variants; why binding and live-virus neutralizing antibody titers to SARS-CoV-2 mRNA vaccines in NSCLC patients were lower than the healthy vaccinees, with significantly lower live-virus neutralization of Delta and Omicron variant compared to the wild-type strain; why cancer patients appear vulnerable to SARS-CoV-2 infection and have poor outcomes. It is clear that the TNFα–NF-κB pathway is one of important pathways to control the progression of variant SARS-CoV-2 and lung cancer. NF-κB inhibitors suppress the endogenous ACE2 at both transcriptional (mRNA) and translational (protein) levels in human lung cancer cells (Lee et al., 2021). Our present results have demonstrated that TBrC and T significantly inhibits both the growth of human lung cancer A549 and H460 cell lines as well as the TNFα-induced nuclear transcriptional activation of NF-κB p65 in A549 cells without affecting cell viability of the normal human lung cells (Figure 5), suggesting that TBrC and T of tea could display more benefits *in vivo* than *in vitro* against SARS-CoV-2 damage to human health. Therefore, the regulation of host NF-κB pathway could be another important mechanism of TBrC inhibiting SARS-CoV-2. This may also explain why some anticancer drugs and natural products can be used to kill both cancer and virus. For example, anticancer drug masitinib significantly reduced viral titers in the lungs and nose and lung inflammation in mice, and was effective in vitro against multiple variants (Drayman et al., 2021). Moreover, RAS pathway and microtubulin inhibitor rigosertib has been investigated in phase I/IIa clinical trials for KRAS+ NSCLC patients (ClinicalTrials.gov Identifier: NCT04263090). The anti-SARS-CoV-2 study of rigosertib has been in preclinic phase (Onconova Therapeutics Inc., 2022 Updated).

More effective drugs are urgently needed now for the pandemic and in the future. Tea as an anticancer natural product has just been found to be effective against SARS-CoV-2 viruses (Chowdhury and Barooah, 2020; Liu et al., 2021; Ohgitani et al., 2021). T is an amino acid occurring naturally in tea leaves and in smaller amount in other plants. As a dietary supplement, it is known to be safe and provides a wide range of health benefits. We semi-synthesized TBrC by using T with the goal of developing T's bioactivity. The fluorescence visualization of TBrC in mice could facilitate the efficacy and mechanistic studies of TBrC. When coupled with SARS-CoV-2-specific targeting neutralizing antibody, TBrC may be used simultaneously as a therapeutic agent and a non-invasive diagnostic/evaluation agent. *In silico* analysis indicated that TBrC showed similar binding modes with masitinib and nirmatrelvir against M^pro^/3CL as well as MLN-4760 against ACE2. More importantly, the potential activity of TBrC against mutant SARS-CoV-2 infection and replication as well as the molecular mechanisms of action could exhibit the potential advantages of TBrC over the antiviral vaccine and/or neutralizing antibody of SARS-CoV-2, which are shown in the flow chart of anti-SARS-CoV-2 of TBrC (Figure 6). Due to the bioactivity of TBrC mentioned above and the roles of ACE2-Spike and M^pro^/3CL as well as NF-κB p65 during the infection and replication of the wildtype and mutants Delta and Omicron SARS-CoV-2 as well as the easily detectable fluorescent imaging of TBrC *in vivo*, TBrC has the potential for further research and development in clinical application as anti-SARS-CoV-2 drug candidate, especially for lung cancer patients with SARS-CoV-2. The data from this study facilitate the discovery and translation of TBrC as an effective treatment for patients infected with SARS-CoV-2.

## 5 Lead contact

Further information should be requested from the Lead Contact, Erxi Wu (Erxi.Wu@BSWHealth.org, 1-254-724-3785).

## Declarations

### Author contribution statement

Jing Lu; Ying Zhang & Dan Qi: Performed the experiments; Analyzed and interpreted the data; Wrote the paper.

Chunyan Yan; Benhao Wu & Jason H.Huang: Analyzed and interpreted the data; Wrote the paper.

Jianwen Yao; Erxi Wu & Guoying Zhang: Conceived and designed the experiments; Contributed reagents, materials, analysis tools or data; Wrote the paper.

### Funding statement

This work was supported by Shandong Provincial Natural Science Foundation to Guoying Zhang (ZR2019MH076; 20092009GG10002087), the National Natural Science Foundation of China (81603024; 30973553), National Key Research and Development Program of China (2017YFB0702600, 2017YFB0702602, 2017YFB0702602-2), the Ministry of Science and Technology of the People’s Republic of China to Guoying Zhang (“863 grant”, 2012AA020206), the Science and Technology Support Program for Youth Innovation in Universities of Shandong (2020KJM003) and Taishan Scholar Project (Jing Lu), and Corbett Estate Fund for Cancer Research to Erxi Wu (62285-531021-41800; 62285-531021-51800; 62285-531021-61800; 62285-531021-71800)

### Data availability statement

Data included in article/supp. material/referenced in article.

### Declaration of interest’s statement

The authors declare no conflict of interest.

### Additional information

No additional information is available for this paper.
